# Practical Departments

**Published:** 1905-07-15

**Authors:** 


### PRACTICAL DEPARTMENTS.

### APPARATUS FOR HEATING AND COOKING BY ELECTRICITY.

The Prometheus Company's Appliances.

Efficiency demands that all the electricity shall be converted into heat-waves of that length which will have the greatest distributing power. The British Prometheus Company, of Kingston-on-Thames, who have been working at the problem for a number of years, claim to have secured this in their apparatus by means of a special form of conductor; and they also claim, by means of air-jackets and other arrangements, to largely prevent the heat generated from being delivered where it is not wanted. Connection can be made to any supply system by arranging sockets on the walls, or in any convenient position. The vessels intended for heating liquids, keeping food warm, and cooking generally are fitted with air-jackets outside the heating elements, with the result that, air having a high thermal resistance, there are smaller losses by convection currents in the air surrounding the vessel than would otherwise be the case. The Prometheus Company claim that from 90 per cent. to 97 per cent. of the heat delivered by the electric current finds its way into the liquid or other substance to be heated.

There are many processes where heat is used in cooking, and also in warming rooms, where, when once a certain temperature has been reached, a much smaller quantity of heat will maintain it. For this purpose the Prometheus Company have worked out a very simple form of self-indicating regulator. The apparatus is arranged on the same lines as the controllers of electric tramcars, but in place of the handle used by the driver, the user of a Prometheus electrical heating apparatus simply slips the regulator off, turns it round to the figure required—half or quarter heat—and slips it on again, the operation taking less time than it does to read the instructions for doing it. The Prometheus Company have worked out a very large number of apparatus for all kinds of heating. In addition to the domestic cooking utensils mentioned, which can stand on a sitting-room or hospital-ward table—they make grills, hot-plates, hot-cupboards, roasting-ovens, water and milk-heaters in various forms, mugs, jugs, etc., egg boilers, apparatus for heating liquids by immersing heated conductors in the liquids, bed and foot-warmers, sterilisers for milk and for surgical instruments, flat irons for laundry work, goffering machines, etc.; and they also make a varied assortment of apparatus for heating rooms, to be placed in the fireplace or in any part of the room and to glow (incandescent lamps being used in these apparatus), or to present merely a hot surface capable of setting up convection currents. The Promethus Company state that, with electricity at 1½d. per Board of Trade unit, one of their apparatus will heat a room 12 feet by 14 feet by 10 feet for nine hours at an average cost of 1d. per hour.

### THE "TORPEDO" WASHING MACHINE.

A novel form of washing machine that has, we understand, only been constructed in small sizes up to the present, is that known as the "Torpedo," made by Mr. Joseph Shaw, of Huddersfield. The novelty consists in the fact that compressed air takes the place of steam, and that the air is compressed in the working of the apparatus. The machine consists of a central hollow cylinder with two conical ends, one of which is removable and forms the opening for the entrance of the clothes. The whole apparatus is slung on trunnions, like a cannon, the trunnions being fixed in the centre of the cylindrical portion, and it is worked to and fro, on the trunnions, by hand or power, an oscillating motion being given. To work the machine, it is placed in the position with its movable end up. The conical end is removed, the clothes, water, and soap are introduced, and the conical cover replaced. Necessarily there is a space between the top of the clothes and the apex of the movable cover, containing only air. When the machine is oscillated, the air in this space is compressed, the clothes acting as a piston, and a certain amount of heat is generated by the act of compression. The compression of the air, aided by the heat and by the oscillation, cause the dirt to be disturbed, as in the steam washing machines, and the soapy solution to penetrate to all parts of the clothes.

